# Untargeted metabolomic analysis of ischemic injury in human umbilical vein endothelial cells reveals the involvement of arginine metabolism

**DOI:** 10.1186/s12986-023-00737-0

**Published:** 2023-03-30

**Authors:** Ruihao Wu, Jiayin Zhong, Lei Song, Min Zhang, Lulu Chen, Li Zhang, Zhaohui Qiu

**Affiliations:** 1 Department of Cardiovascular Medicine, Tongren Hospital, Shanghai Jiao Tong University School of Medicine, No. 1111, Xianxia Road, Changning District, Shanghai, 200336 China; 2 Hongqiao International Institute of Medicine, Tongren Hospital, Shanghai Jiao Tong University School of Medicine, Shanghai, 200336 China

**Keywords:** Untargeted metabolomics, Human umbilical vein endothelial cells, Glucose oxygen deprivation, Arginine metabolism, Metabolic proteins

## Abstract

**Objective:**

In this study, differentially expressed metabolites of vascular endothelial cells were examined to further understand the metabolic regulation of ischemic injury by untargeted metabolomics.

**Method:**

Human umbilical vein endothelial cells (HUVECs) were selected to construct an ischemia model using oxygen–glucose deprivation (OGD) and 0, 3, 6, and 9 h of treatment. After that, cell survival levels were determined by CCK8 detection. Flow cytometry, ROS detection, JC-1 detection, and western blotting were used to measure apoptosis and oxidative stress in cells. Then, combined with UPLC Orbitrap/MS, we verified the impacted metabolism pathways by western blotting and RT‒PCR.

**Results:**

CCK8 assays showed that the survival of HUVECs was decreased with OGD treatment. Flow cytometry and the expression of cleaved caspase 3 showed that the apoptosis levels of HUVECs increased following OGD treatment. The ROS and JC-1 results further suggested that oxidative stress injury was aggravated. Then, combined with the heatmap, KEGG and IPA, we found that arginine metabolism was differentially altered during different periods of OGD treatment. Furthermore, the expression of four arginine metabolism-related proteins, ASS1, ARG2, ODC1 and SAT1, was found to change during treatment.

**Conclusion:**

Arginine metabolism pathway-related proteins were significantly altered by OGD treatment, which suggests that they may have a potential role in ischemic injury.

**Supplementary Information:**

The online version contains supplementary material available at 10.1186/s12986-023-00737-0.

## Introduction

Acute myocardial infarction (AMI) is myocardial necrosis caused by acute and continuous ischemia and hypoxia of coronary arteries, which can be complicated by shock, arrhythmia, or heart failure. According to the research data of the Atherosclerosis Risk In Communities (ARIC) of National Heart Lung and Blood Institute (NHLBI) from 2005 to 2014, there are 605,000 new cases of myocardial infarction and 200,000 recurrent cases every year [1], and the proportion of young patients hospitalized for AMI has increased, especially among women [2]. From the perspective of etiology, AMI is usually due to the rupture or erosion of unstable plaques with high lipid contents and easy breakage after coronary atherosclerosis, resulting in thrombosis and lumen blockage, which lead to certain degrees of ischemia and irreversible myocardial injury [3].

Cardiomyocytes have always been the focus of AMI research. However, as an important gateway for material exchange between cardiomyocytes and blood, the injury state and functionality of vascular cells after blockage have always been ignored. After ischemia, vascular endothelial cells first develop edema, which involves the production of reactive oxygen species (ROS) and stimulates a series of oxidative stress and apoptosis processes, followed by vasomotor dysfunction, microcirculation disorder and vascular rupture [4–6]. Previous studies have mainly focused on the effects of drug interventions on oxidative stress in endothelial cells caused by hypoxia [7], but little attention has been given to metabolism. With the increase in metabolism-related research, using a new method to explore the metabolic changes in vessels, especially vascular endothelial cells, will reach a new stage for the study of myocardial infarction.

Untargeted metabolomics is a discipline that was newly developed after genomics and proteomics. It is a new technology that specializes in systematic and high-throughput analyses of changes in metabolite components. Metabolomics is usually defined as a complete set of metabolites or small molecular chemicals found and analyzed in a given organelle, cell, organ, biological fluid or organism [8, 9]. Among many metabolic analysis techniques, high-resolution mass spectrometry (HRMS) technology, including Orbitrap or time-of-flight systems, has developed rapidly. Through these systems, researchers can analyze the changes in more extensive biological metabolites without limiting the types of internal and external samples and obtain sufficient metabolic data with the minimum sample size; however, the complexity of this method imposes higher requirements on operators and analysts [10]. Therefore, many technologies, including HRMS, have promoted the progress of cardiovascular disease metabolism-related research. For instance, phenylalanine metabolism, sphingolipid metabolism, and glycolipid metabolism were reported to be seriously disturbed after AMI [11]. Some of these metabolites have been proven to be related to the pathological changes of AMI, such as upregulation of eicosatrienoic acid and eicostetraenoic acid, suggesting an inflammatory response [12]. Under normal circumstances, due to the low content of mitochondria in vascular endothelial cells, 85% of ATP is still produced by glycolysis [13]. The production capacity of glutamine oxidation and fatty acid oxidation is used to compensate for the tricarboxylic acid cycle [14, 15]. However, as the first threshold of the vascular wall, the existing research on the metabolic changes in vascular endothelial cells in acute myocardial infarction is still insufficient.

It is necessary to explore the cellular or molecular changes in vascular endothelial cells under the action of different injury-stimulating factors, which is also a new concept for understanding a complex disease. Previous related studies have mostly discussed the effects of exogenous factors, such as some chemicals, on the growth state, structure, and function of endothelial cells induced by injury [16, 17] and have paid little attention to the adaptive and self-regulation processes of endothelial cells in response to injury. In this study, we used an oxygen–glucose deprivation (OGD) cell model to simulate the ischemic and hypoxic environment under myocardial infarction in vitro, and human umbilical vein endothelial cells (HUVECs) were selected as the research subjects to represent the vascular endothelium. Combined with UPLC-Orbitrap/MS, we focused on the changes in endothelial cell metabolic activity after different periods of OGD treatment (0, 3, 6 and 9 h).

To better carry out this study, we attempted to elucidate the self-adaptation and injury regulation processes of vascular endothelial cells under stimulation by ischemia and hypoxia in terms of metabolism by using untargeted metabolomics and further explored the expression of key regulatory proteins by western blotting and RT‒PCR. The significance of this project will not only be in determining the metabolic pathways that play an essential role in the responses of vascular endothelial cells to ischemic environments but also in trying to link and understand the differential metabolism reflected by metabonomics results and functional proteins related to metabolism; these studies are conducive to determining innovative targets and directions for future research.

## Materials and methods

### Cell culture and OGD treatment

HUVECs were acquired from ScienCell Research Laboratories (San Diego, USA). The cells were cultured in endothelial cell medium (ECM) (ScienCell Research Laboratories) containing 5% (v/v) fetal bovine serum (FBS) and 1% penicillin/streptomycin (P/S) at 37 °C in an atmosphere with 5% (v/v) CO_2_ and 95% humidity. The cells were randomly divided into the following four groups: 0, 3, 6, and 9 h OGD-treated groups. The 0 h OGD treatment group was also called the normal control group (NC group). Before cell passage, the culture media was replaced every 3 days, and the cells were split at 70–80% confluence using 0.05% trypsin–EDTA. For the cells in the OGD treatment groups, glucose-free ECM (ScienCell Research Laboratories) containing 1% FBS was used to replace the previous ECM complete culture medium, and the cells were transferred to a three gas incubator and cultured for different periods with gas parameter settings of 1% O_2_, 5% CO_2_ and 94% N_2_.

### Cell viability assay

The viability of HUVECs after different OGD treatment periods was detected with CCK8 reagent (Beyotime, Shanghai). Cells were inoculated on 96-well plates for culture, and six secondary wells were used in each group. One hour before each OGD treatment group reached the predetermined treatment times (3, 6 and 9 h), the orifice plates were removed from the incubator, 10 µl of CCK8 reagent was added to each well and mixed evenly, and the cells in the group were returned to the three air incubator for incubation for 1 h. After reaching the set hypoxia time, the cells were removed from the instrument to measure and record the OD values. Each OGD treatment group was examined with a parallel NC group.

### Detection of cellular ROS

HUVECs were inoculated in 6-well plates for culture and divided into four groups (NC group and 3-, 6- and 9-h OGD treatment groups). A ROS detection reagent (Beyotime, Shanghai) was prepared in advance; DCFH-DA was diluted with serum-free culture solution at a ratio of 1:1000 so that the final concentration was 10 μmol/L. After each group reached the predetermined treatment time, the culture medium in each well was discarded, and the cells were washed twice with PBS. Then, an appropriate volume of diluted DCFH-DA was added to each well and left to stand at 37 °C for 20 min. After reaching the predefined incubation time, the ROS fluorescence signal intensities of the cells were measured under a fluorescence microscope (× 200 field), and photographs were taken. This process used a 525 nm emission fluorescence wavelength and 488 nm excitation fluorescence wavelength (green light).

### Flow cytometry

HUVECs were seeded onto 6-well plates at a density of 80,000 cells per well. After 24 h, cells were subjected to the corresponding ODG treatments associated with the above groups and cultured in a conventional 37℃ incubator or three gas incubator for a predetermined time. Then, the cells were harvested using trypsin (Gibco, USA), and subsequently incubated with Annexin V-FITC and propidium iodide (PI) (Beyotime, China) for 20 min in the dark; then, cells were transferred to an ice bath. The apoptosis rates of the HUVECs were determined by a FACSCanto II (BD Biosciences, USA). Flow Jo 10.0 was used to analyze the flow cytometry results.

### Mitochondrial membrane potential (MMP) detection

The MMP values were detected with a JC-1 probe (Beyotime, Shanghai) based on the manufacturer’s instructions. In brief, HUVECs were evenly seeded on six-well plates, and when the cell density reached 70%, they were grouped and treated accordingly. Following treatment, the cells were incubated with JC-1 solution for 20 min at 37 °C. The JC-1 solution is a working solution that was prepared in advance by diluting JC-1 (200X) into ultrapure water and JC-1 buffer. After incubation, the cells were washed twice with JC-1 buffer (1X); then, the samples were observed and images were captured under a fluorescence microscope (× 100 field).

### Western blotting

HUVECs treated with OGD for different times (0, 3, 6 and 9 h) were used as samples for western blotting. The samples were homogenized and lysed with RIPA buffer (Epizyme, Shanghai) containing 1% sodium deoxycholate, 1% Triton X-100, and 0.1% SDS with protease and phosphatase inhibitor mixtures for 20 min on ice. The lysates were centrifuged at 12,000 × rpm at 4 °C for 25 min, and the supernatants were separated into cell extract mixtures. Next, the total protein concentrations were measured using a bicinchoninic acid assay (Beyotime, Shanghai). For western blotting analyses, the extracted protein samples were separated on 7.5–12.5% SDS‒PAGE gels (Epizyme, Shanghai) and transferred onto nitrocellulose membranes (Immobilon, USA). The membranes were incubated overnight at 4 °C with primary antibodies, followed by horseradish peroxidase-conjugated secondary antibodies (Cell Signaling Technology). Then, ECL developer (Epizyme, Shanghai) was used to soak the membranes in the dark. Immunoreactive signals were detected using a Tanon-5200 analyzer (Shanghai, China).

The following antibodies were used for western blotting: rabbit monoclonal anti-cleaved caspase 3 (Asp175) antibody (#9664S, Cell Signaling Technology, 1:1000); mouse monoclonal anti-β-actin antibody (#3700S, Cell Signaling Technology, 1:1000); rabbit monoclonal anti-GAPDH antibody (#5174S, Cell Signaling Technology, 1:1000); rabbit monoclonal anti-ASS1 (#70720S, Cell Signaling Technology, 1:1000); rabbit monoclonal anti-SAT1 (#61586S, Cell Signaling Technology, 1:1000); rabbit monoclonal anti-ODC1 antibody (ab270268, Abcam, 1:1000); rabbit polyclonal anti-ARG2 antibody (ab264066, Abcam, 1:1000); goat anti-rabbit IgG antibody (ab6721, Abcam, 1:5000); and goat anti-mouse IgG antibody (ab6789, Abcam, 1:5000).

### Metabolite sample extraction

Cells were inoculated in 6-cm culture dishes, and 12 culture dishes were used for each group. After inoculation, cells were cultured in an incubator at 37 °C for 24 h, and the corresponding OGD treatments were administered according to the group division. Then, the cells were harvested and quenched by the liquid nitrogen contact method. One milliliter of a precooled mixture of methanol and water (4:1, V/V) was added to each dish. The cells were fully scraped with a cell scraper and transferred to 1.5-ml centrifuge tubes, which were sealed with sealing film and stored at -80 °C for metabolomic analysis. Before all samples were officially tested on the instrument, equal volumes (approximately 20 µL) of each sample were taken as quality control (QC) samples for mixing.

### UPLC-Orbitrap/MS

The UPLC system was combined with an Orbitrap/MS ion trap mass analyzer (Waters Corp, USA) equipped with an electrospray ionization source. It was operated in either positive or negative ionization mode using 70,000 mass resolution at 200 m/z. Additionally, we used data-dependent (dd-MS2, TopN = 10) MS/MS mode with a full scan mass resolution of 17,000 at 200 m*/z*. The chromatographic column we used was a Waters HSS T3 C18 column (2.1 × 100 mm, 1.7 µm). The main chromatographic conditions were defined as follows: 2 µL injection volume, 24 °C column temperature, 0.30 ml/min flow rate and mobile phase containing liquid aqueous solution (0.1% formic acid) and liquid b-acetonitrile (0.1% formic acid). The scan range was 150–1,500. After optimization, the chromatographic gradient was as follows: 5% in liquid B in 0–2 min, 5–95% in liquid B in 2–10 min, 95% in liquid B in 10–15 min, and 5% in liquid B in 15–18 min. We acquired data in centroid mode by using Thermo Xcalibur 2.2 software (Thermo Fisher Scientific, USA).

### Metabolomic data analysis

Peak alignment and extraction were performed with Compound Discoverer software (Thermo Fisher Scientific, USA). Then, a data table was constructed containing information about the retention times, *m/z* values, and peak areas. Next, we imported the data into SIMCA-P software version 13.0 (Umetrics, Umea, Sweden) for principal component analysis (PCA) and partial least squares discrimination analysis (PLSDA). PCA was used to assess the overall segregation trend between samples. A supervised PLSDA analysis model was used to screen for significantly differentially expressed metabolites among the OGD treatment groups and the NC group. According to the PLSDA model, we selected the parameters with variable importance in projection (VIP) values > 1.0, and two-tailed Student’s t tests were used to determine the *p* values; the statistical tests were performed by SPSS Statistics 18.0 and *p* < 0.05 was considered statistically significant. To identify the differentially expressed metabolites, accurate ion masses were input into the human metabolome database (HMDB) to match the exact molecular weights, and MS1/MS2 fragment ions were systematically searched. Furthermore, to confirm the metabolite details, we used our internal standard metabolite library for quantification. In addition, pathway enrichment analysis was conducted using the Kyoto Encyclopedia of Genes and Genomes (KEGG) database and MetaboAnalyst 5.0 (https://www.metaboanalyst.ca/). Heatmapping was used to better show the trend changes and internal differences of different metabolites. Finally, metabolite interaction network analysis was conducted by using the ingenuity pathway analysis (IPA) online database.

#### Real-time polymerase chain reaction (RT‒PCR)

We extracted 100 µL of total RNA by precipitation using TRIzol (Beyotime, Shanghai), and the RNA was reverse transcribed to complementary DNA (cDNA) in a specific reverse transcription system (TOYOBO, Japan). Ten microliters of cDNA product from each sample was used as a template to conduct quantitative PCR analysis in an Applied Biosystems QuantStudio™ 6 and 7 Pro (Thermo Fisher Scientific) using SYBR Green PCR master mix (Vazyme, Nanjing) under the following conditions: initial denaturation at 95℃ for 30 s, 40 cycles at 95℃ for 10 s, 60℃ for 30 s, and a dissociation curve at 95℃ for 15 s, 60℃ for 60 s and 95℃ for 15 s. The relative expression levels were calculated using ACTB as an internal control. The primer sequences used are listed in Additional file 1: Table S1.

#### Statistical analysis

GraphPad Prism 5.0 (GraphPad Software) was used for statistical analysis. One-way ANOVA was used for comparisons of more than two groups, with the Newman‒Keuls test used for multiple comparisons. All error bars represent the SEMs. Significance was defined as *p < 0.05, **p < 0.01, and ***p < 0.001. In some results of this study, the superscripts ‘#’, ‘$’ and ‘*’ of individual groups have the same meaning and are only used for differentiation.

## Results

### Different OGD treatment times had different effects on the survival of HUVECs

**Fig. 1:**
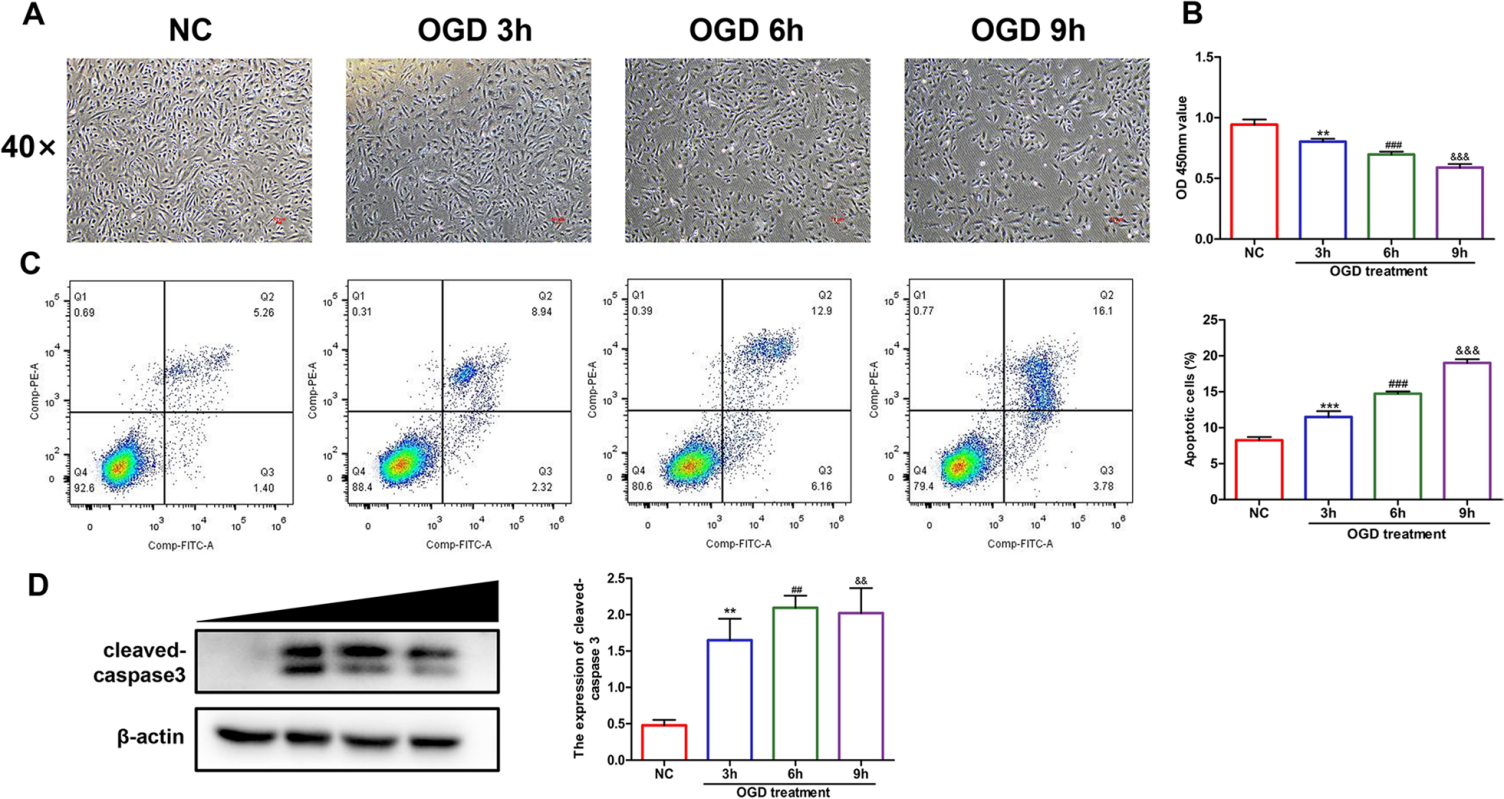
HUVECs treat with OGD over different time periods (0, 3, 6 and 9 h) showing that the degree of apoptosis increases with time. **A** Brightfield (40 × field) observations showed the growth and distribution of HUVECs after different OGD treatment times, n = 3/group. **B** The absorbances (OD values) of each group after different OGD treatment times were measured by CCK8 assays, n = 5/group. **C** Annexin V-FITC/PI reagent was used to detect apoptosis of HUVECs after different OGD treatment times by flow cytometry, n = 3/group. **D** The level of the apoptotic marker protein cleaved Caspase3 was measured by western blotting, n = 3/group. One-way ANOVA with multiple comparisons was utilized to determine the statistical significance as follows: *p value < 0.05, **p value < 0.01, and ***p value < 0.001

First, low magnification brightfield (× 40 field) observations showed that with an extension of treatment time, the number of cells decreased, the morphologies of cells were abnormal and the distributions were scattered (Fig. 1A). The growth state of HUVECs was affected by OGD treatment time. Then, the CCK8 experiment was conducted to measure the changes in cell viability of HUVECs following different OGD treatment times (0, 3, 6 and 9 h). The results are displayed as OD values (Fig. 1B). The longer the OGD treatment time, the more significant the effect on cell viability, suggesting that time is a key factor in OGD-induced HUVEC injury. Flow cytometry was used to detect apoptosis of HUVECs in this study. By comparing the proportions of the sums of early apoptosis and late apoptosis (Q2 + Q3) of the four groups of cells (Fig. 1C), the proportions of cells showing apoptosis under glucose deficiency and hypoxia increased with time. Then, the western blotting results showed that the expression of cleaved Caspase 3 significantly increased after OGD treatment, which further verified that OGD could induce apoptosis to a certain degree (Fig. 1D).

### Different OGD treatment times affected HUVEC apoptosis and oxidative stress to varying degrees

**Fig. 2:**
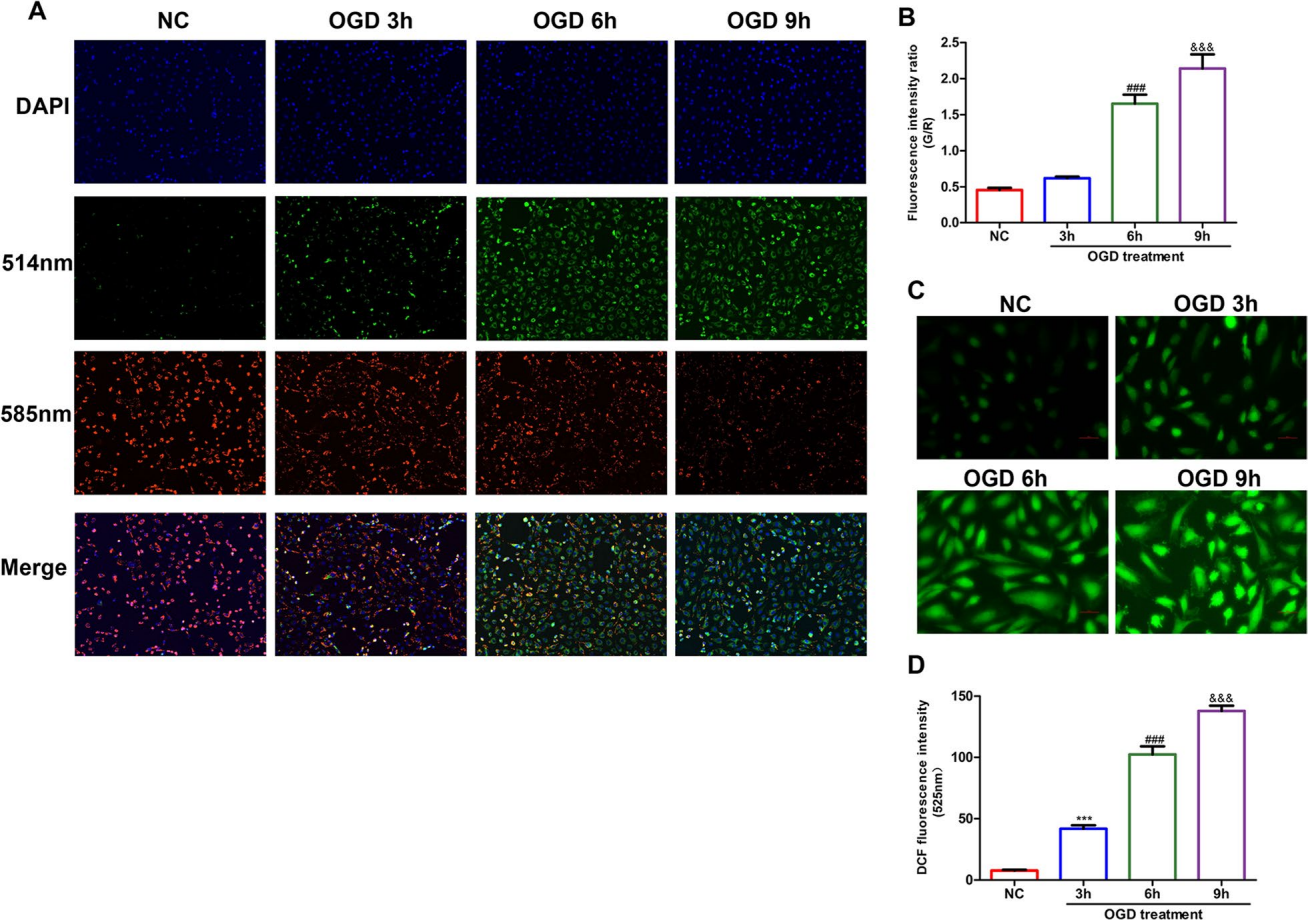
HUVECs showed different levels of oxidative stress and early apoptosis after different times of OGD treatment. **A** The mitochondrial membrane potentials of the four groups of cells were observed by JC-1 detection under 585 nm emission and 514 nm excitation with a fluorescence microscope (× 100 field), n = 3/group. **B** Graphical representation of the green/red fluorescence intensity ratios in JC-1 detection. **C** ROS immunofluorescence assays were used to observe the production of ROS in cells (× 200 field), n = 3/group. **D** Fluorescence intensity value of DCF compound reflecting ROS content. One-way ANOVA with multiple comparisons: *p value < 0.05, **p value < 0.01, and ***p value < 0.001

A decreased mitochondrial membrane potential (Δψm) is also characteristic of the early stage of apoptosis and oxidative stress. JC-1 detection in the four groups of cells showed that the mitochondrial membrane potentials of cells decreased gradually with prolonged OGD treatment (Fig. 2A, B), which revealed the effect of OGD on HUVEC injury. Detection of cellular ROS can effectively indicate the production of ROS in mitochondria due to stimulation by injury and reflect the oxidative stress of cells to a certain extent (Fig. 2C, D). Immunofluorescence photos taken at high magnification (× 200 field) showed ROS production in HUVECs in the NC group and OGD treatment groups at three time periods. The cellular ROS contents increased with an extension of injury time.

### There were significant differences in the composition of intracellular metabolites of HUVECs among the OGD treatment groups at different times

**Fig. 3:**
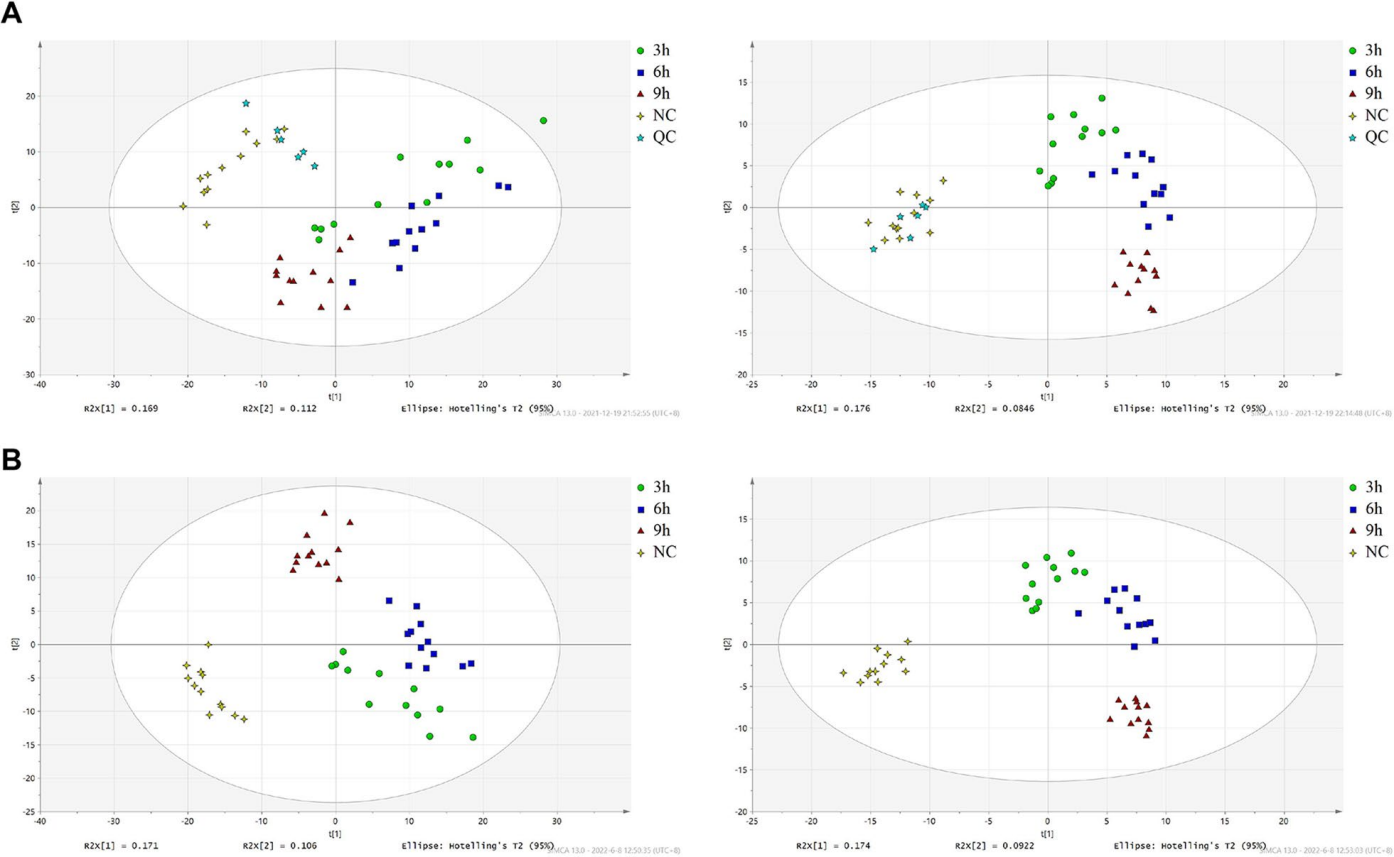
Multivariate statistical analyses of HUVECs treated with OGD at different times based on UHPLC-QTPF/MS data. **A** PCA of each treatment group and NC group. R½[1] X1 = 0.178 and R½[2] X2 = 0.108 in (1) and R½[1] X1 = 0.175 and R½[2] X2 = 0.0932 in (2). **B** PLS-DA of each treatment group and NC group. R½[1] X1 = 0.171 and R½[2] X2 = 0.106 in (1) and R½[1] X1 = 0.174 and R½[2] X2 = 0.0922 in (2). PLS-DA: partial least squares discriminant analysis and PCA: principal component analysis; (1) and (2) represent positive ions (left) and negative ions (right)

The principal component analysis (PCA) and partial least squares discriminant analysis (PLS-DA) modeling methods were performed on results to verify the degree of aggregation between samples (Fig. 3A, B). The PCA results indicated a total of 2 principal components in positive mode with R½[1] X1 = 0.178 and R½[2] X2 = 0.108 and 2 principal components in negative mode with R½[1] X1 = 0.175 and R½[2] X2 = 0.0932. The result of QC also showed reasonable data (Fig. 3A). As a supervised model analysis method, PLS-DA can effectively reflect group differences between samples (Fig. 3B). The results showed 2 principal components in positive mode with R½[1] X1 = 0.171 and R½[2] X2 = 0.106 and 2 principal components in negative mode with R½[1] X1 = 0.174 and R½[2] X2 = 0.0922. Therefore, we can conclude that there are obvious differences in metabolite components among the four groups of samples; that is, after OGD treatments, the metabolic activities of HUVECs may be altered to varying degrees with treatment time. However, this result needs further verification.

**Fig. 4:**
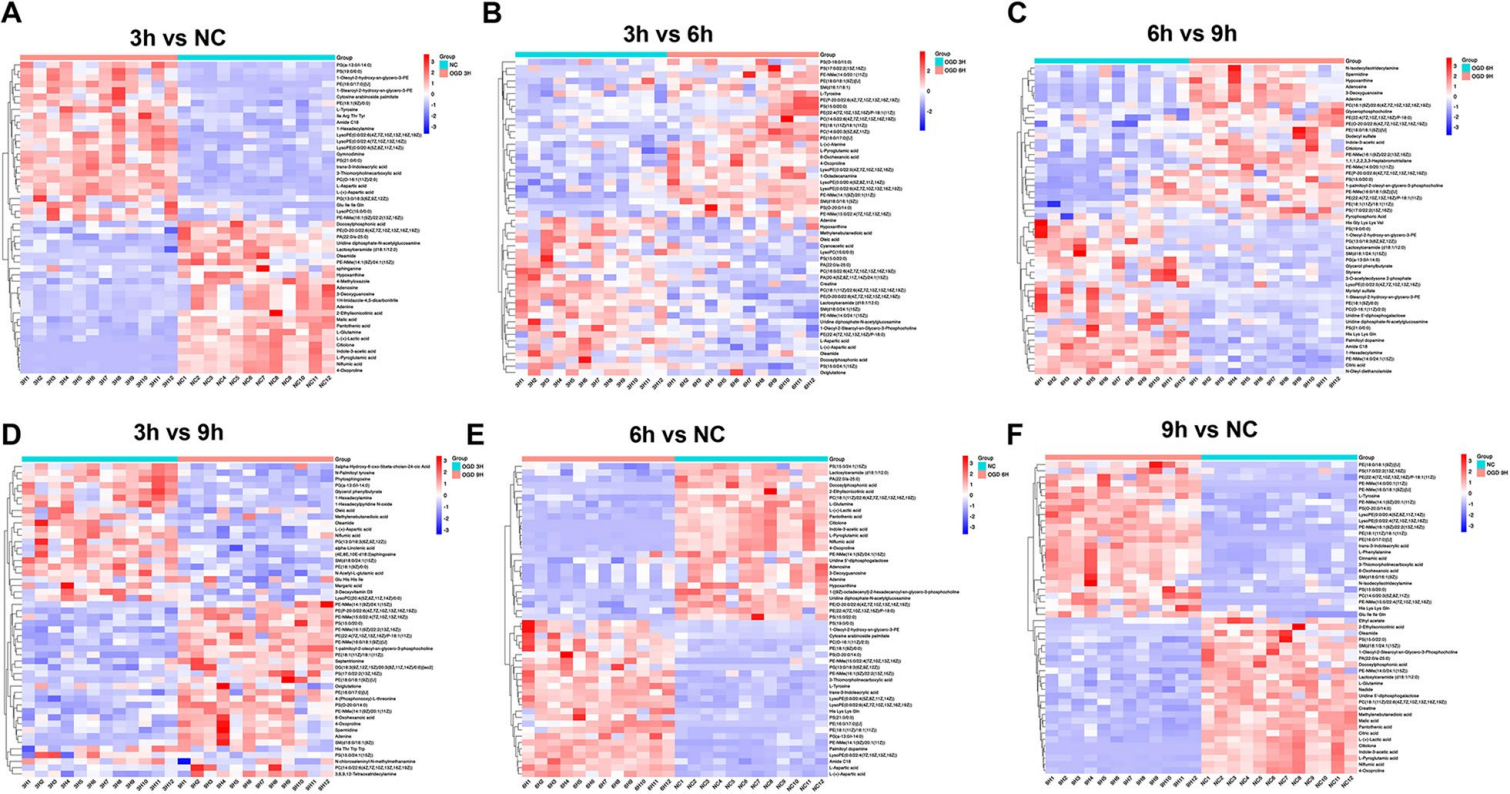
Significant changes in various types of metabolites were identified by comparing the differentially expressed metabolites between the glucose and oxygen deprivation treatment groups at different times in heatmaps. **A** The NC group and the 3-h OGD treatment group. **B** The 3-h OGD treatment group and the 6-h OGD treatment group. **C** The 6-h OGD treatment group and the 9-h OGD treatment group. **D** The 3-h OGD treatment group and the 9-h OGD treatment group. **E** The NC group and the 6-h OGD treatment group. **F** The NC group and the 9-h OGD treatment group. In the pairwise comparison, only the top 25 differentially up- and down-regulated metabolites were selected for heatmaps to observe the distribution of metabolite types

A heatmap of the pairwise comparisons can intuitively reflect the changes in metabolites among groups. For the comparison of each group, we selected the top 50 differentially increased and decreased metabolites for the heatmap display to analyze the trend of changes in metabolite levels (Fig. 4). By analyzing these 50 differentially expressed metabolites, we determined the production stages of some key metabolic molecules in the OGD treatment process. Most phospholipids, especially phosphatidyl ethanolamine, showed upward trends with the extension of injury time throughout the OGD treatment process (Fig. 4A–C). The production level of lysophosphatide ethanolamine also showed an increasing trend (Fig. 4A). In the early stage of OGD treatment (< 6 h), the amount of tyrosine increased significantly, and the production of L-aspartic acid increased first and then decreased; meanwhile, L-glutamine showed a downward trend early in injury, and this change lasted until the late stage of damage treatment (Fig. 4C). The production levels of some small molecules related to energy metabolism, such as adenosine, hypoxanthine and adenine, maintained downward trends before 6 h of injury and exhibited slight increases thereafter; meanwhile, creatine decreased throughout the whole process (Fig. 4A–C). However, further data are needed to determine which key differential metabolic molecules show significant differences from the NC group throughout the entire OGD treatment process; this result will be of great significance because it will identify the metabolic pathways that are closely related to OGD injury.

### The contents of 52 intracellular metabolites changed significantly after OGD treatment of HUVECs

**Fig. 5:**
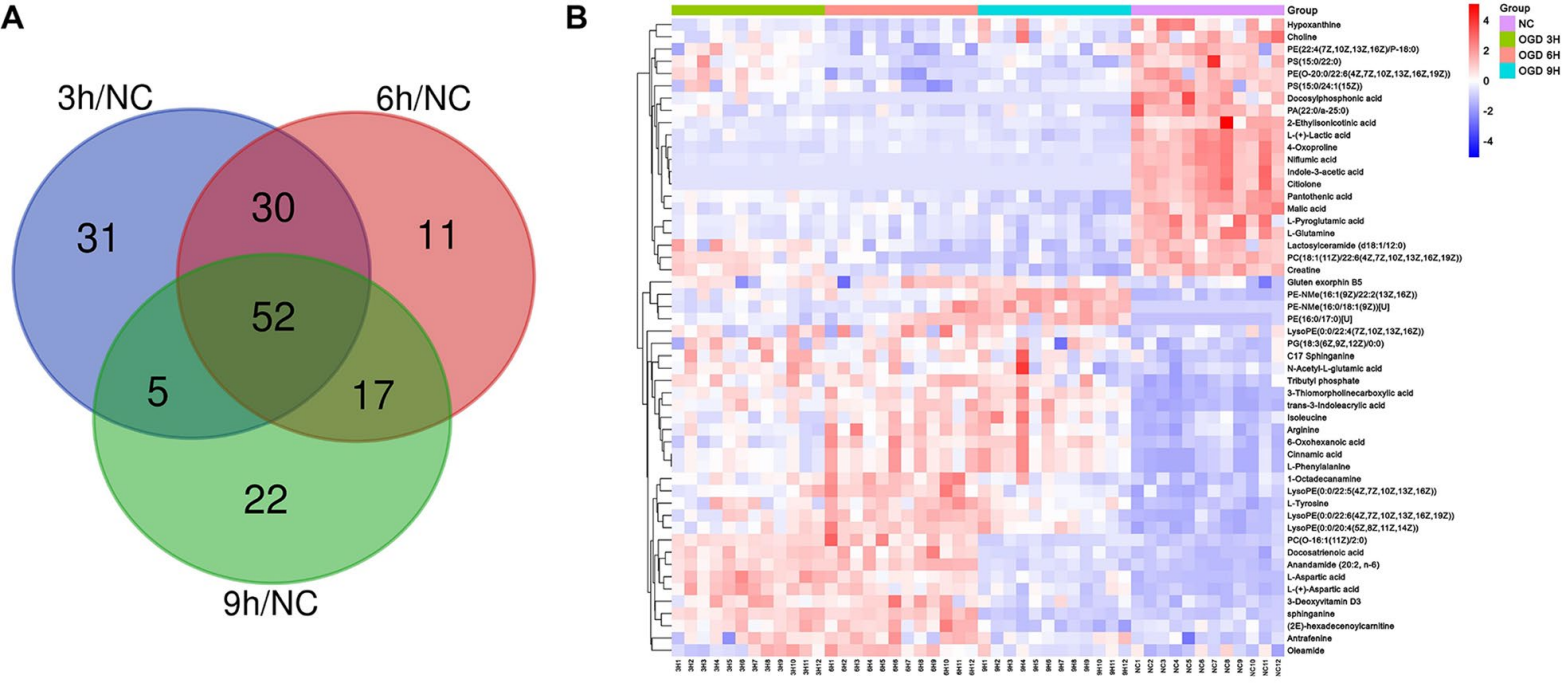
Fifty-two different metabolites were obtained by data screening, and the trends in their levels were further observed. **A** The metabolites with significantly different expression among the OGD treatment groups and the NC group were analyzed with a Venn diagram. **B** Heatmap of 52 significantly changed endogenous metabolites in the NC group and the OGD treatment group at different times

**Fig. 6:**
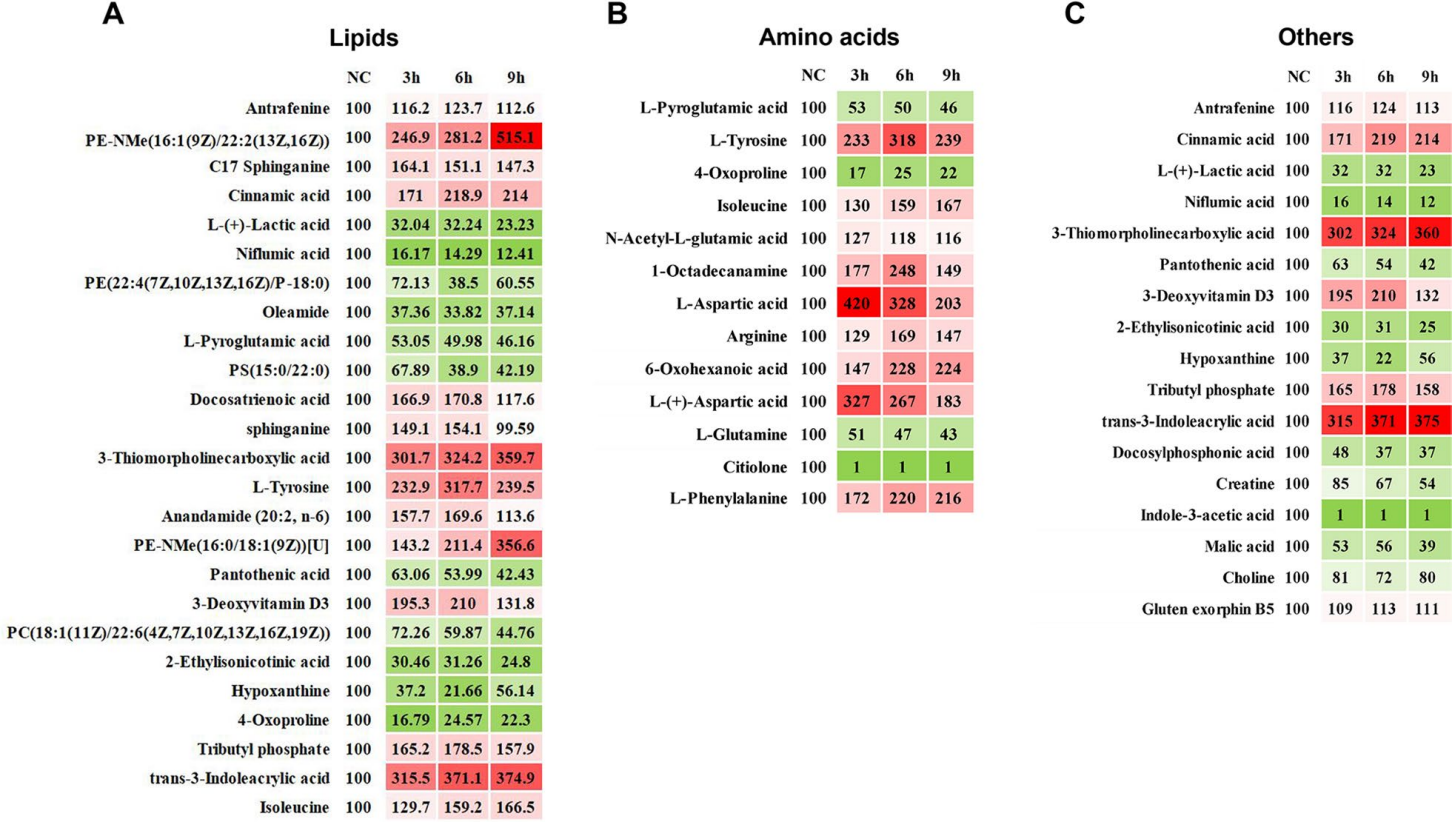
The thermogram distinguished the 52 metabolites and quantified the relative differences between the groups. **A** Trends of lipid metabolites. **B** Trends of amino acid metabolites. **C** Trends of other small molecule metabolites. “100” of the NC group is the standardized value

By using a Venn diagram, the data for the NC group and the injury treatment groups in each time period that were obtained by the untargeted metabolomics can be collectively analyzed, and 52 metabolites were identified by determining the intersections of differentially expressed metabolites (Fig. 5A). These metabolites showed significant differences among the NC group and OGD treatment groups for the three time periods (Table 1), and these substances exhibited apparent changes in the early to late stages of OGD treatment in the experiment and did not return to levels close to normal. The specific metabolites and corresponding HMDB-related information are listed in Table 1. The heatmap results for the 52 metabolites from the four groups also better exhibited the changes in their overall trends with extension of OGD processing time (Fig. 5B). Next, lipids and amino acids were sorted separately by using the categories defined in the HMDB (Fig. 6). A total of 22 lipids and 13 amino acids were identified, and most of them showed trends that increased with time from injury. However, among the lipids, phosphatidylserines such as PS (15:0/22:0) and phosphatidyl acids such as PA (22:0/a-25:0) decreased significantly, and most phosphatidylethanolamines showed upward trends as described above, except for a few such as PE (22:4 (7z, 10z, 13z, 16Z)/p-18:0). Lipid-related products, including endogenous cannabinoids such as anandamide (20:2, n-6), increased before OGD treatment for 6 h and then decreased, and another similar substance, oleamide, decreased significantly after injury (Fig. 6A). Although amino acids account for a small proportion of the 52 metabolites, the amount of many functionally important amino acids changed significantly during the injury process. L-glutamine decreased significantly after injury, while arginine, L-tyrosine and L-aspartic acid increased in the early stage of injury and decreased slightly in the late stage (Fig. 6B). Among other small metabolic molecules, L-( +)-lactic acid and pantothenic acid remained at low levels after OGD treatment, and creatine showed a steady downward trend with extension of treatment time; meanwhile, hypoxanthine showed a slight upward trend after 6 h compared with a low level found in the previous OGD treatment period (Fig. 6C).

**Table 1:** Specific information of 52 different metabolites and statistical data compared among groups

HMDB ID	Metabolites	Chemical Formula	OGD 3 h (campared to NC)		OGD 6 h (campared to NC)		OGD 9 h (campared to NC)	
			VIP	*P* value	VIP	*P* value	VIP	*P* value
HMDB0113077	PE-NMe(16:1(9Z)/22:2(13Z,16Z))	C_44_H_82_NO_8_P	1.70289	1.92592E−05	1.66745	2.28916E−07	2.30093	4.6024E−15
HMDB00269	C17 Sphinganine	C_18_H_39_NO_2_	1.46233	2.352E−04	1.33258	3.065E−04	1.21892	0.014
HMDB0000567	Cinnamic acid	C_9_H_8_O_2_	1.86402	1.52399E−07	1.73518	2.1238E−08	2.04107	6.44925E−08
HMDB0000190	L-( +)-Lactic acid	C_3_H_6_O_3_	2.11509	2.50911E−11	1.90061	1.22435E−11	1.88508	1.83479E−12
HMDB15573	Niflumic acid	C_13_H_9_F_3_N_2_O_2_	2.00148	7.47579E−11	1.83636	4.87347E−11	2.21157	3.30513E−11
HMDB0009610	PE(22:4(7Z,10Z,13Z,16Z)/P-18:0)	C_45_H_82_NO_7_P	1.04598	0.023	1.56711	9.03504E−06	1.27268	7.476E−04
HMDB0002117	Oleamide	C_18_H_35_NO	1.34176	0.001	1.41106	1.044E−04	1.48937	9.962E−04
HMDB0000267	L-Pyroglutamic acid	C_5_H_7_NO_3_	2.04281	3.49203E−12	1.85208	1.460E−11	2.22692	1.42635E−11
HMDB0112334	PS(15:0/22:0)	C_43_H_84_NO_10_P	1.10491	0.018	1.5416	1.64053E−05	1.41924	8.97162E−05
HMDB0002823	Docosatrienoic acid	C_22_H_38_O_2_	2.04249	2.65503E−12	1.74053	1.537E−08	1.58928	3.396E−04
HMDB00269	sphinganine	C_18_H_39_NO_2_	2.030	3.50456E−12	1.87921	8.040E−13	1.43512	0.002
HMDB0059611	3-Thiomorpholinecarboxylic acid	C_5_H_9_NO_2_S	1.98806	3.120E−10	1.77605	2.80179E−09	2.16666	4.06858E−10
HMDB0000158	L-Tyrosine	C_9_H_11_NO_3_	1.6261	2.06472E−05	1.7731	2.57191E−09	2.13575	1.94702E−09
HMDB0004080	Anandamide (20:2, n-6)	C_22_H_37_NO_2_	2.0825	2.07568E−14	1.900	5.860E−14	1.7336	5.00426E−05
HMDB0010569	PE-NMe(16:0/18:1(9Z))[U]	C_40_H_78_NO_8_P	1.20726	0.005	1.07189	0.008	2.23574	5.44715E−12
HMDB0000210	Pantothenic acid	C_9_H_17_NO_5_	1.99642	1.1488E−10	1.85432	1.0905E−11	2.25273	3.8706E−12
HMDB0257271	3-Deoxyvitamin D3	C_27_H_38_O_3_	1.75864	1.22095E−06	1.5958	1.958E−06	1.20133	0.013
HMDB0008090	PC(18:1(11Z)/22:6(4Z,7Z,10Z,13Z,16Z,19Z))	C_48_H_82_NO_8_P	1.51545	3.410E−04	1.86638	1.550E−10	1.87125	6.82929E−12
HMDB14747	2-Ethylisonicotinic acid	C_8_H_10_N_2_S	1.50266	1.659E−04	1.36405	1.837E−04	1.72477	6.447E−05
HMDB0000157	Hypoxanthine	C_5_H_4_N_4_O	1.47417	2.384E−04	1.47948	2.51303E−05	1.15849	0.018
HMDB0304793	4-Oxoproline	C_5_H_6_NO_3_	2.11805	1.96164E−11	1.86463	1.75681E−10	1.83714	1.08157E−10
HMDB0259164	Tributyl phosphate	C_12_H_27_O_4_P	1.71615	2.520E−06	1.72378	2.56868E−08	1.73184	6.6567E−05
HMDB0015488	Antrafenine	C_30_H_26_F_6_N_4_O_2_	1.26498	0.005	1.44279	9.860E−05	1.10069	0.005
HMDB0000734	trans-3-Indoleacrylic acid	C_11_H_9_NO_2_	1.99701	4.86916E−10	1.85511	1.47929E−11	2.210	3.000E−11
HMDB0028910	Isoleucine	C_12_H_24_N_2_O_3_	1.2761	3.382E−03	1.59195	2.35788E−06	1.67513	1.282E−04
HMDB0001138	N-Acetyl-L-glutamic acid	C_7_H_11_NO_5_	1.79597	3.7027E−06	1.27394	0.001	1.8452	9.45557E−06
HMDB0029586	1-Octadecanamine	C_18_H_39_N	1.710	2.80175E−06	1.72933	2.15626E−08	1.29633	0.006
HMDB0000191	L-Aspartic acid	C_4_H_7_NO_4_	2.15264	1.549E−12	1.92564	1.36645E−12	1.64639	6.14935E−07
HMDB0011493	LysoPE(0:0/22:4(7Z,10Z,13Z,16Z))	C_27_H_48_NO_7_P	1.78406	4.391E−07	1.900	8.5147E−14	2.140	1.53227E−09
HMDB0009243	PE(O-20:0/22:6(4Z,7Z,10Z,13Z,16Z,19Z))	C_47_H_82_NO_8_P	1.920	1.76082E−07	1.71293	1.90797E−07	1.58694	2.91321E−06
HMDB0004866	Lactosylceramide (d18:1/12:0)	C_42_H_79_NO_13_	1.57092	1.715E−04	1.57817	7.55103E−06	1.69056	1.398E−07
/	Docosylphosphonic acid	/	1.56857	4.535E−05	1.60114	1.5028E−06	1.93659	1.50443E−06
HMDB0000064	Creatine	C_4_H_9_N_3_O_2_	1.40158	5.148E−04	1.76162	5.00067E−09	2.240	8.62628E−12
HMDB0253127	(2E)-hexadecenoylcarnitine	C_23_H_43_NO_4_	1.66186	8.090E−06	1.57845	2.720E−06	1.02879	0.035
HMDB0251511	Arginine	C_6_H_14_N_4_O_2_	1.53586	3.530E−04	1.68193	1.62749E−07	1.740	4.43055E−05
HMDB0010662	PG(18:3(6Z,9Z,12Z)/0:0)	C_42_H_77_O_10_P	1.400	7.086E−04	1.42783	6.591E−05	1.25316	8.898E−03
HMDB0011496	LysoPE(0:0/22:6(4Z,7Z,10Z,13Z,16Z,19Z))	C_27_H_44_NO_7_P	1.7615	1.06164E−06	1.86598	4.13293E−12	1.89127	2.91103E−06
HMDB0303320	6-Oxohexanoic acid	C6H11NO3	1.430	0.001	1.740	1.02059E−07	1.74617	1.61092E−08
HMDB0304946	Indole-3-acetic acid	C_12_H_13_NO_2_	2.0012	9.2389E−11	1.82885	9.10133E−11	2.19543	9.14134E−11
HMDB0112341	PS(15:0/24:1(15Z))	C_45_H_86_NO_10_P	1.26641	6.263E−03	1.48342	5.397E−05	1.49756	2.162E−05
HMDB0008949	PE(16:0/17:0)[U]	C_37_H_74_NO_7_P	1.94426	2.57888E−08	1.57179	3.26879E−06	2.16404	4.68133E−10
HMDB0000191	L-( +)-Aspartic acid	C_4_H_7_NO_4_	2.03892	1.34506E−11	1.805	4.24244E−10	1.7339	8.90807E−05
HMDB0000156	Malic acid	C_4_H_6_O_5_	2.0371	2.77918E−09	1.78053	1.85329E−08	1.86592	1.38296E−11
/	PC(O-16:1(11Z)/2:0)	/	2.10827	9.564E−17	1.68483	1.371E−07	1.25484	0.008
HMDB0115667	PA(22:0/a-25:0)	C_50_H_99_O_8_P	1.87776	5.210E−07	1.79838	6.83898E−09	1.72528	3.85648E−08
HMDB0000097	Choline	C_5_H_14_NO	1.25932	0.004	1.40849	9.15523E−05	1.22327	0.016
HMDB0000641	L-Glutamine	C_5_H_10_N_2_O_3_	1.89853	1.30733E−08	1.760	5.97735E−09	2.120	3.5229E−09
HMDB0059795	Gluten exorphin B5	C_30_H_38_N_6_O_11_	1.01634	0.028	1.03318	0.012	1.17445	0.002
HMDB0011487	LysoPE(0:0/20:4(5Z,8Z,11Z,14Z))	C_25_H_44_NO_7_P	1.780	4.515E−07	1.86795	5.18856E−12	1.95136	7.51815E−07
HMDB0011494	LysoPE(0:0/22:5(4Z,7Z,10Z,13Z,16Z))	C_27_H_46_NO_7_P	1.56903	4.46706E−05	1.73288	1.7063E−08	1.52629	6.760E−04
HMDB0250301	Citiolone	C_6_H_9_NO_2_S	1.96733	6.38225E−10	1.79882	6.35378E−10	2.16223	6.37351E−10
HMDB0000159	L-Phenylalanine	C_9_H_11_NO_2_	1.86684	1.38922E−07	1.73517	2.121E−08	2.04383	5.90069E−08

OGD, Oxygen–glucose deprivation; VIP, Variable important in projection; NC, normal control group

### Many metabolites, including L-arginine, have potential effects on the dysfunction and regulation of HUVECs in OGD

**Fig. 7:**
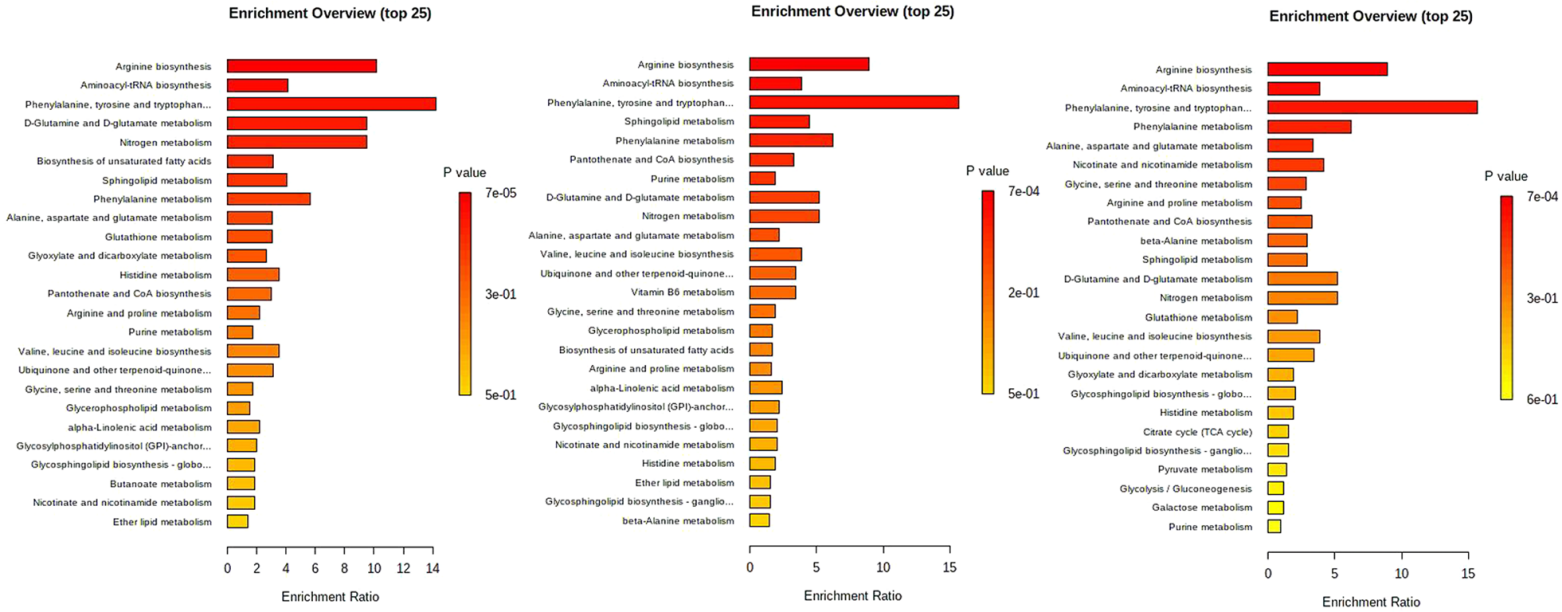
KEGG enrichment analysis bar charts of enriched metabolism pathways among the OGD groups and NC group. **A** Comparison between the OGD 3 h group and the NC group. **B** Comparison between the OGD 6 h group and the NC group. **C** Comparison between the OGD 9 h group and the NC group

**Fig. 8:**
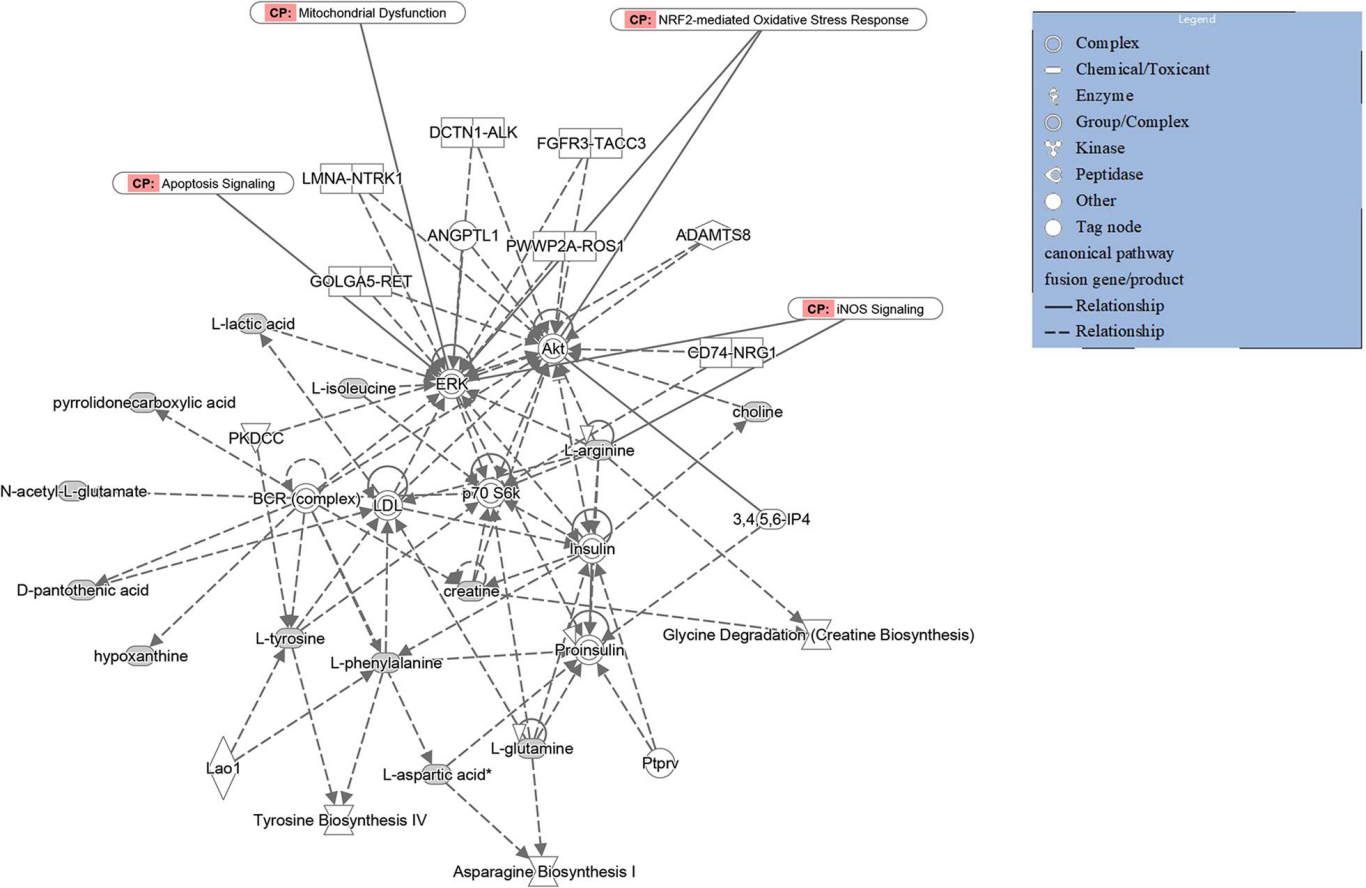
IPA of the correlations among the 52 differentially expressed metabolites in the cell death mechanism. CP: canonical pathway; full line: direct relation; dotted line: indirect relation

According to the bar chart showing the KEGG enrichment analysis results, there were significant differences in metabolic pathways among the three OGD treatment groups and the NC group; furthermore, most of the top changes were amino acid metabolism, especially arginine biosynthesis metabolism (Fig. 7A–C). The IPA method was utilized to further explore the correlations among the 52 metabolites, HUVEC damage, and the self-regulation pathway after OGD treatment to identify the metabolites that played key roles (Fig. 8). From the IPA results, various selected metabolites, mainly amino acids, had more intuitive direct or indirect relationships with apoptosis, mitochondrial dysfunction, Nrf2-mediated oxidative stress, and inducible nitric oxide synthase (iNOS) activation. Among these metabolites, arginine, glutamine, N-acetyl-L-glutamic acid, phenylalanine, tyrosine, isoleucine, and aspartic acid are likely to play predictable roles by affecting ERK, Akt, P70S6K and other key signal transduction pathways to some extent. In addition, important small molecule metabolites, such as creatine, lactic acid, hypoxanthine, and choline, also participate in these regulatory processes.

**Fig. 9:**
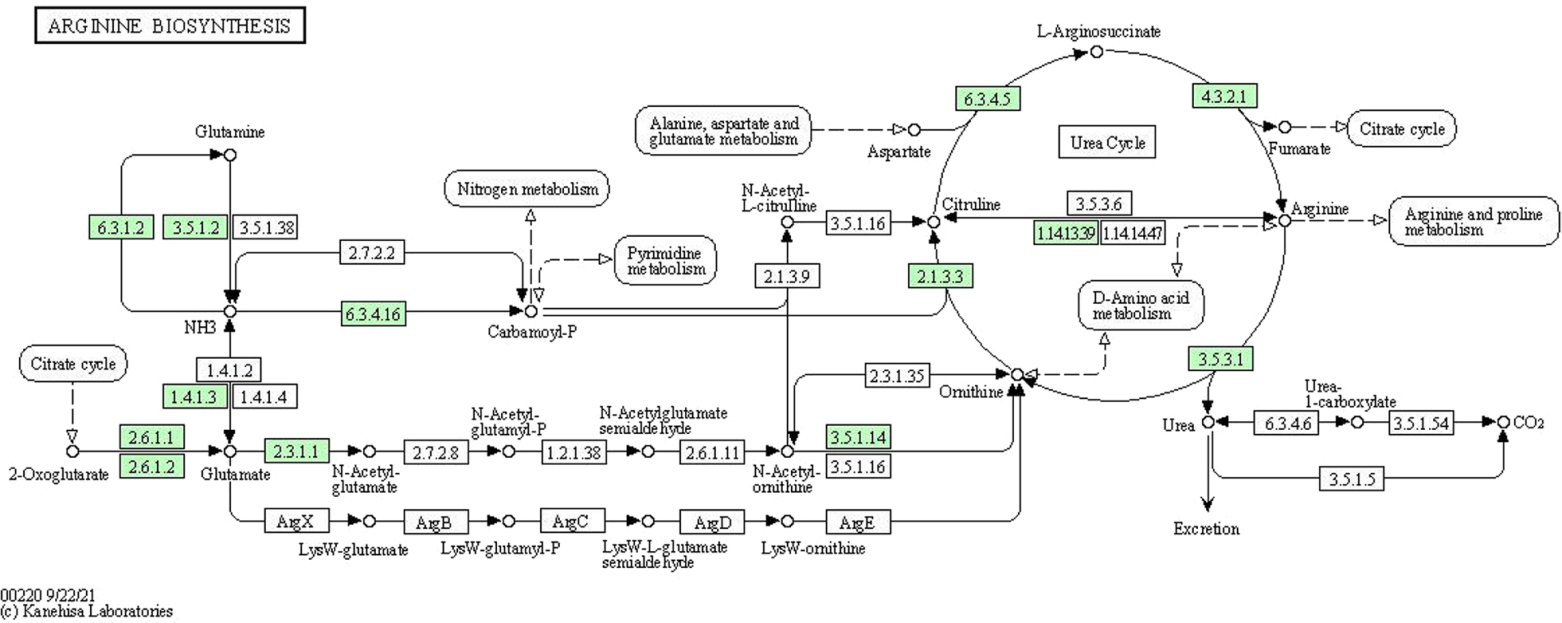
KEGG analysis results for the arginine metabolism pathway

The KEGG enrichment analysis showed that the arginine metabolism pathway had an essential effect on the entire OGD injury process, and there were significant differences among the three treatment period groups (3, 6 and 9 h) and the NC group (Fig. 9). In this pathway, arginine is an important node in the ornithine cycle, also known as the urea cycle. Arginine generates ornithine and urea under the action of arginase. Ornithine reacts with carbamyl phosphate to generate citrulline, which promotes the nitrogen excretion process of the body. At the same time, various amino acids can also transfer amino groups to oxobutanedioic acid through transamination and participate in the urea cycle in the form of aspartic acid; α-ketoglutarate is also a key component of the citric acid cycle. Therefore, arginine metabolism is involved in various metabolic pathways of cells, and changes in arginine metabolism are of great value to the study of cell injury models.

### OGD treatment affected the expression of some key proteins in the arginine metabolic pathway and polyamine synthesis in HUVECs

**Fig. 10:**
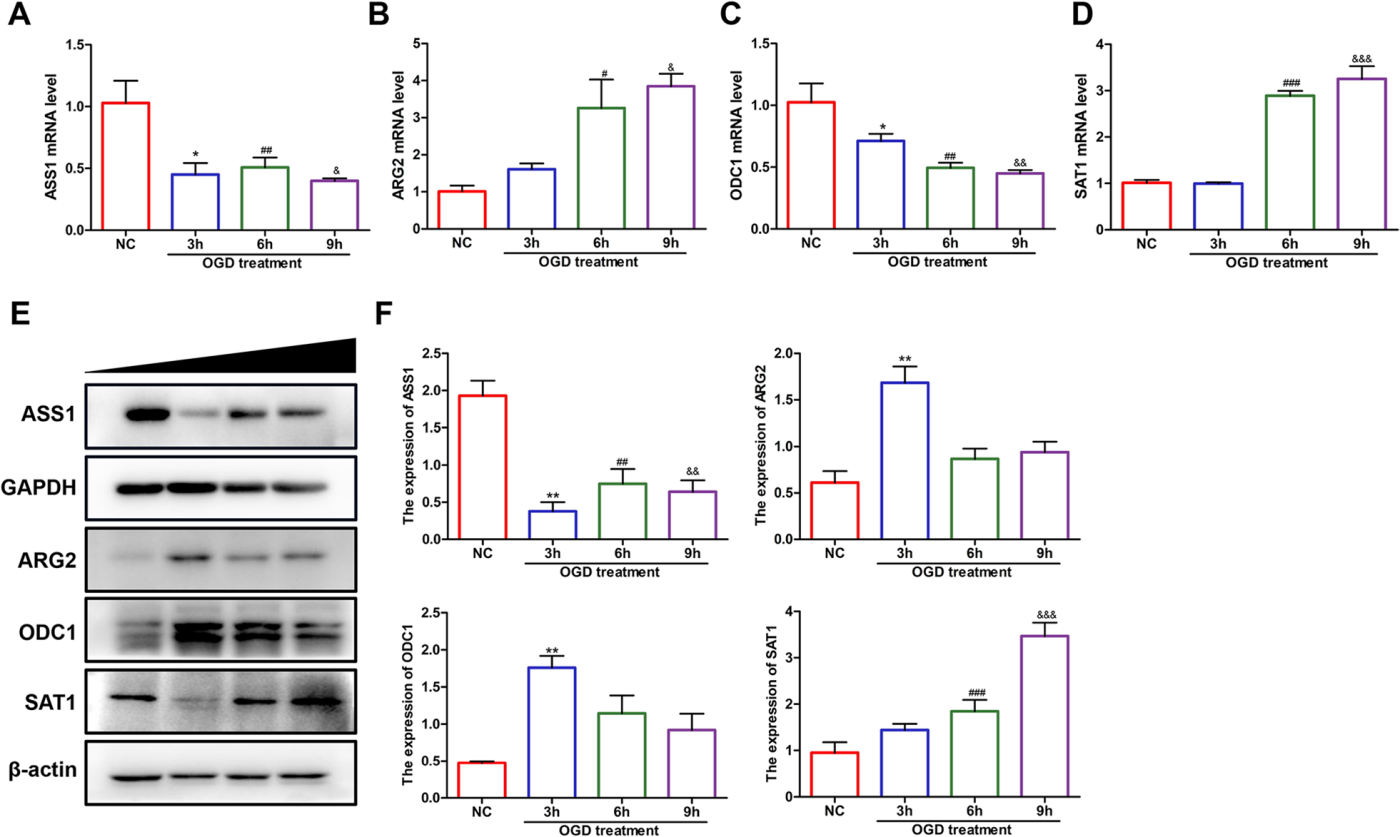
Arginine metabolism pathway-related proteins changed significantly in HUVECs after different OGD treatment periods (0, 3, 6 and 9 h). **A**–**D** Intracellular mRNA levels of ASS1, ARG2, ODC1 and SAT1 were measured by RT‒PCR, n = 3/group. **E** Western blotting results of four metabolism-related proteins (ASS1, ARG2, ODC1 and SAT1), n = 3/group. **F** Quantitative analysis results of the four proteins. One-way ANOVA with multiple comparisons was utilized to determine the statistical significance as follows: *p value < 0.05, **p value < 0.01, and ***p value < 0.001

RT‒PCR can directly detect the mRNA levels of related proteins in cells, thus indicating changes in protein synthesis. RT‒PCR was used to detect the mRNA synthesis of ASS1 (argininosuccinate synthetase 1), ARG2 (arginase 2), SAT1 (spermidine-spermine acetyltransferase 1) and ODC1 (ornithine decarboxylase 1) in HUVECs after OGD treatment (Fig. 10) at different times. The expression of ASS1 and ODC1 significantly decreased after hypoxia and glucose deficiency (Fig. 10A, B), while the expression of ARG2 and SAT1 increased (Fig. 10C, D). The content changes of four intracellular proteins related to arginine metabolism and polyamine synthesis, ASS1, ARG2, ODC1 and SAT1, were verified by western blotting (Fig. 10E, F). After OGD treatment of cells, the protein content of ASS1 decreased, and ODC1 showed a transient increase at 3 h after injury treatment and then showed a decreasing trend. The SAT1 contents significantly increased in the later stage of treatment, while ARG2 increased in the early stage and decreased in the later stage. These results suggest that arginine-related metabolic pathways, especially the polyamine synthesis pathway, may play an important potential role in the effects of OGD-mediated glucose oxygen deprivation on cells.

## Discussion

Acute myocardial infarction (AMI) is an ischemic disease caused by acute blockage of coronary arteries. In fact, it not only causes necrosis of myocardial cells but also damages cardiac vascular cells to varying degrees under the influence of ischemic factors [4, 18]. However, vascular endothelial cells have received little attention in the study of cardiac ischemic injury. In addition to the basic functions of barrier and permeability [19], vascular endothelial cells also secrete factors that regulate vasomotor and functional homeostasis, such as nitric oxide (NO) and endothelin-1 [20–23]. In recent years, the direction of metabolic correlation has become a new research focus in the cardiovascular field [24, 25]. Lee et al. found that IFN-γ regulates tryptophan catabolism to disrupt glucose metabolism in endothelial cells and leads to an increased transfer of metabolism to fatty acid oxidation [26]. Earlier, Kerstin et al. found that the transcription factor FoxO1 can regulate cell proliferation and vascular expansion by regulating glucose metabolism and energy metabolism in endothelial cells [27]. However, the current research on metabolic and functional changes of vascular endothelial cells is still insufficient. Since HUVECs are equivalent and consistent with arterial endothelial cells [28] and using these materials is more feasible, they are often used in the cardiovascular field to study many characteristics of coronary endothelial cells and are widely recognized. Therefore, in this study, HUVECs were chosen as the research object to simulate the ischemic conditions of the vascular endothelium through the glucose and oxygen deprivation model (OGD), and the successful establishment of the model was verified through CCK8, ROS detection, flow cytometry, JC-1 detection and the detection of the apoptosis-related protein cleaved caspase 3 concentration; this study provides a basis for future metabolism-related research.

Metabolism has been proven to affect cell proliferation, apoptosis, oxidative stress, inflammation and autophagy to a large extent [29, 30]. Before this, Chouchani et al. found that the selective accumulation of succinic acid in the tricarboxylic acid cycle (TCA) during ischemia‒reperfusion (I/R) is a key link leading to ROS production, which may be a new mechanism of oxidative stress damage caused by I/R [31]. Another metabolite, butyrate, can pass through the PPARδ/Mir-181b pathway to reduce NOX2 expression and ROS production in vascular endothelial cells, thereby preventing endothelial dysfunction in atherosclerosis [32]. Due to the high energy consumption of the heart [33], explorations of the mechanisms related to cardiovascular metabolism often focus on energy metabolism and glucose and lipid metabolism [34]. In recent years, researchers have begun to realize that amino acid metabolism plays an important role in the cardiovascular system [35]. One study demonstrated that endogenous glutamate drives local calcium release (LCR) in sinoatrial node pacing cells, adding a potentially important factor to the coupled clock theory that explains the origin of spontaneous firing [36]. Li's research team found that accumulations of branched chain amino acids (BCAAs) inhibited glucose metabolism by inhibiting the activity of the pyruvate dehydrogenase complex (PDH) and made the heart sensitive to ischemic injury [37]. In terms of blood vessels, L-arginine can improve endothelial cell dysfunction and vasomotor dysfunction through nitric oxide synthase (NOS)-mediated production of NO [38]. In addition, one study pointed out that the mechanism of vascular endothelial injury in coronary heart disease (CHD) with type 2 diabetes and chromosome 1q25 variations may be related to γ-glutamyl cycle impairment [39]. However, further exploration of cardiovascular metabolism needs to be combined with more advanced and newer technologies.

Metabolomics enables us to measure thousands of metabolites in biological tissues, cells or body fluids, which can greatly promote the development of metabolic research and the understanding and diagnosis of complex diseases [40]. Andreas et al. found that the activities of polyamine metabolites were increased in patients with impaired left ventricular ejection fraction by using untargeted metabolomics technology, which allowed the effective detection of heart failure in patients with a reduced ejection fraction [41]. In addition, a targeted LC‒MS-based metabolomic study of plasma found that changes in several metabolites, including certain amino acids, pyrimidine metabolites and pentose phosphate pathway metabolites, were observed as early as 10 min after the planned infarction [42]. Yang's team also found through LC/MS metabolomics that serum BCAA levels were significantly higher in CAD patients than in healthy individuals and were independent of other traditional risk factors, which also added a reliable basis for conducting extensive cardiovascular research on BCAAs [43]. In this study, UPLC Orbitrap/MS was used to analyze the changes in intracellular metabolic components of HUVECs after different OGD treatment periods and identify significant changes and different types of metabolic substances through Venn diagram analysis and heatmap trend analysis. We then combined the KEGG and IPA analysis methods and found that arginine metabolism and its related pathways may play a potential role in the endothelial cell ischemia model.

The core of arginine metabolism is the urea cycle (also known as the ornithine cycle), which is the key link of nitrogen metabolism. In endothelial cells, arginine synthesizes nitric oxide through endothelial nitric oxide synthase (eNOS) and plays a central role in regulating blood flow and maintaining the integrity of endothelial cells through the NO-sGC (soluble guanylate cyclase)-cGMP pathway [44, 45]. Studies have shown that eNOS deficiency is associated with severe left ventricular dysfunction [46]. However, in ischemic heart failure, leukocyte iNOS (inducible type) activation promotes local inflammation and cardiac remodeling [47]. Arginine produces NO through the classic NOS pathway to exert physiological effects, and some studies have also demonstrated that arginine-related functional regulation may have other important pathways. According to the study of Wang et al., arginine can reinternalize CD36 into the nuclear endosome through the mTOR-vATPase axis to prevent lipid accumulation [48]. In addition, L-arginine supplementation for 10 weeks was found to significantly improve the cardiac recovery of patients with heart failure and improve their quality of life [49]. In addition, polyamines, downstream products of the arginine metabolic pathway, have been shown to affect cell survival and proliferation. For example, spermidine can increase carnosine phosphorylation, prevent myocardial hypertrophy and decrease diastolic function, thus delaying the progression of heart failure [50]. In this study, a change in the arginine pathway was found in an ischemic environment. The results were not limited to a specific metabolite but focused on the proteins in the pathway to find a new research target.

ASS1, ARG2, ODC1 and SAT1 are indispensable regulatory proteins related to arginine metabolism. ASS1 is a key protein that promotes the conversion of citrulline to arginine succinate in the urea cycle. A recent study indicated that ASS1 plays a central role in antimicrobial defense by controlling inflammatory macrophage activation through cellular citrulline depletion [51]. In this study, ASS1 expression was downregulated, which suggested that ASS1 might have a potential role in endothelial cell injury caused by ischemia. Another protein observed to be downregulated was ODC1, which regulates polyamine synthesis in cells and is downstream of arginine metabolism. In our results, ODC1 levels did not change significantly in the early stage of glucose oxygen deprivation and even slightly increased but there was a significant decrease after 6 h of injury. It is speculated that the changes in this protein in the early stage may be regulated by some ‘buffering’ mechanism, which still needs further exploration. In oncology, it is thought that inhibiting the activity of ODC1 can regulate the synthesis of polyamines and thus inhibit the proliferation of tumor cells [52]. This regulatory mechanism of cell proliferation has great reference value in the cardiovascular field. Compared with ARG1, ARG2 is mainly expressed in extrahepatic tissues [53], so it has become the focus of this research. The results showed that ARG2 expression transiently increased in the early stage of injury and gradually decreased in the later stage. This trend is slightly similar to that of ODC1. We speculate that this may be caused by the acute stress response in the early stage which induces the synthesis of polyamines with protective effects. In addition, it has been reported that ARG2 participates in the downregulation of IL-10-mediated inflammatory mediators such as succinic acid, hypoxia inducible Factor 1α (HIF-1α), and IL-1-β in vitro [54]; these mediators can also be a good starting point for future research. Finally, SAT1 was upregulated in the later stage of glucose oxygen deprivation-treated endothelial cells. This suggests that SAT1 may damage vascular endothelial cells under ischemia to some extent. Therefore, in this study, we confirmed abnormal changes in the arginine metabolism pathway and the polyamine synthesis pathway of vascular endothelial cells under glucose oxygen deprivation.

The above results provide a new direction for future research and will help transition from the study of metabolism to exploring the mechanism of action of metabolism-related proteins. Of course, this part of the work also benefited from the implementation of metabolomics and successful screening and analysis, from which we can identified the direction of cardiovascular metabolism research that needs to be addressed next. Although untargeted metabolomics still cannot provide information regarding the changes in spatial distributions of intracellular metabolites, we still want to know the metabolic pathway changes at the subcellular level; studies on spatial distributions will be a next step.

## Conclusion

In this research, a vascular endothelial cell ischemia model was established under glucose oxygen deprivation. By using UPLC Orbitrap/MS, differentially expressed metabolites were identified. Focusing on arginine-related pathways, the study found that some key proteins in this metabolism pathway changed significantly with the extension of injury time, thus providing a new understanding of the arginine metabolism pathway; specifically, the important role of metabolism-related proteins in cell injury was identified. This study will provide a good research basis to further explore the regulatory mechanism of vascular endothelial cells and other cardiovascular diseases at the molecular level.

## Supplementary Information

**Additional file 1: Table S1**. Primer sequences used for RT-PCR; **Figure S1**. (A) TIC (pos). (B) TIC (neg), TIC: total ion flow chromatogram; pos: positive; neg: negative.

## Data Availability

Data available on request.

## Abbreviations

AMI: Acute myocardial infarction
HRMS: High-resolution mass spectrometry
HUVECs: Human umbilical vein endothelial cells
NC: Normal control group
OGD: Oxygen–glucose deprivation
ECM: Endothelial cell medium
ROS: Reactive oxygen species
PCA: Principal component analysis
PLSDA: Partial least squares discriminant analysis
UPLC Orbitrap/MS: Ultra-high performance liquid chromatography coupled with orbitrap mass spectrometry
HMDB: The Human Metabolome Database
KEGG: Kyoto Encyclopedia of Genes and Genomes
IPA: Ingenuity Pathway Analysis
ASS1: Argininosuccinate synthetase 1
ARG2: Arginase 2
ODC1: Ornithine decarboxylase 1
SAT1: Spermidine-spermine acetyltransferase 1
