# Supplementary material for: Untargeted metabolomic analysis of ischemic injury in human umbilical vein endothelial cells reveals the involvement of arginine metabolism
Source: Nutr Metab (Lond). 2023 Mar 30;20:17. doi: 10.1186/s12986-023-00737-0 (PMC10061785; doi:10.1186/s12986-023-00737-0)
Supplement: Supplementary file 1 — Additional file 1: Table S1. Primer sequences used for RT-PCR; Figure S1. (A) TIC (pos). (B) TIC (neg), TIC: total ion flow chromatogram; pos: positive; neg: negative. [file 12986_2023_737_MOESM1_ESM.docx]

***Supplementary Material***

**Untargeted metabolomic analysis of ischemic injury in human umbilical vein endothelial cells reveals the involvement of arginine metabolism**

**Supplementary Table1. Primer sequences used for RT-PCR**

| **Gene** | **Primer Sequence (5’ to 3’ )** |
| --- | --- |
| **For human** | |
| ASS1 | F: CTTGGGGCCAAAAAGGTGTTC;  R: GAGGTAGCGGTCCTCATACAG |
| ARG2 | F: TGACATCAACACACCCCTTACC;  R: GTCCACGTCTCTCAGACCAAT |
| ODC1 | F: GTTTTGCGGATTGCCACTGAT;  R: GCTCTTTCGCCCGTTCCAA |
| SAT1 | F: ACCCGTGGATTGGCAAGTTAT;  R: TGCAACCTGGCTTAGATTCTTC |
| ACTB | F: CATGTACGTTGCTATCCAGGC;  R: CTCCTTAATGTCACGCACGAT |


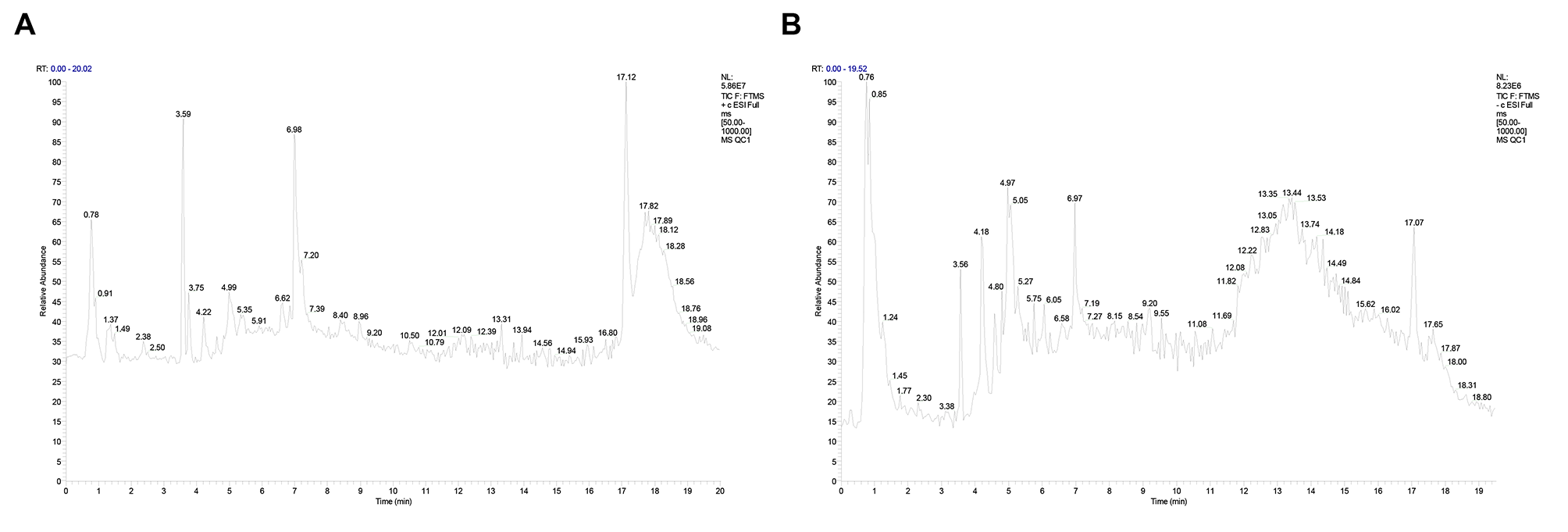


**Supplementary Figure 1.** (A) TIC (pos). (B) TIC (neg), TIC: total ion flow chromatogram; pos: positive; neg: negative.
